# Wilson's Disease: Diagnosis of Wilson's Disease in Ethiopian Young Sisters

**DOI:** 10.1155/2020/7650170

**Published:** 2020-07-24

**Authors:** Nebiyu Bekele, Frew Ewnetu, Tigest Hailu, Zerubabel Tegegne, Abilo Tadesse

**Affiliations:** ^1^Department of Internal Medicine, College of Medicine and Health Sciences, University of Gondar, Gondar, Ethiopia; ^2^Department of Radiology, College of Medicine and Health Sciences, University of Gondar, Gondar, Ethiopia

## Abstract

**Background:**

Wilson's disease is an inherited autosomal recessive disorder of copper metabolism. Clinical signs, biochemical parameters, histologic findings, and/or ATP7B genetic testing are required to diagnose Wilson's disease. *Case Presentation*. 25-year-old and 22-year-old young women (siblings) presented to the University of Gondar Hospital, Northwest Ethiopia, with difficulty of keeping balance of 3-year duration and progressive extremity weakness of 5-year duration, respectively. Both siblings had visible ocular Kayser–Fleischer rings, low serum ceruloplasmin level and increased urinary copper content, ultrasound-evidenced cirrhotic liver disease, and axial T_2_-weighted MRI hyperintensities in basal ganglia, thalamus, and brainstem (midbrain and pons). Diagnosis of Wilson's disease was established in both patients using a diagnostic scoring system proposed by “8^th^ International Meeting on Wilson Disease and Menkes Disease, Leipzig (2001).” Treatment with D-penicillamine as a chelator and zinc sulphate as a metalothionein-inductor was started. Screening of their family members was recommended.

**Conclusion:**

Wilson's disease, declared to be an orphan disease, requires clinical acumen of physicians and expensive investigation modalities for prompt recognition and is inaccessible as required, lifelong drugs for treatment.

## 1. Background

Wilson's disease (WD) was first described by Kinnear Wilson in 1912, as “hepatolenticular degeneration” [1]. It is an inherited autosomal recessive disorder, caused by a mutation in the ATP7B gene, leading to impaired hepatocellular copper transport, and subsequently results in deleterious accumulation of copper in the liver, brain, kidney, and cornea. The prevalence of WD is estimated to be 1 in 30,000 individuals in nonconsanguineous families [2–4].

The clinical features may vary from asymptomatic state with biochemical abnormalities, hepatic diseases, neurologic deficits, and psychiatric disorders [2–11]. Diagnosis is established by a clinical scoring system proposed by “8^th^ International Meeting on Wilson Disease and Menkes Disease, Leipzig (2001)” as presenting clinical and laboratory parameters lack diagnostic accuracy [6]. Symptoms of Wilson's disease can be effectively controlled by medical therapy (chelation therapy, zinc salts, and dietary advice), while liver transplantation is held in reserve for those with severe liver disease and incapacitating neurologic deficit [2–5, 11–14]. Here, we discourse two cases of Wilson's disease presented with neurologic deficits.

## 2. Case Presentation

### 2.1. Case-1

A 25-year-old young woman presented to the University of Gondar Hospital, Northwest Ethiopia, with progressive difficulty of keeping balance of three-year duration and change in voice (slurring of speech) and tremor of hands of six-month duration. She had no visual disturbance, weakness of extremities, or bladder or bowel problem. She had no memory loss or epileptic fits. Her younger sister has been wheel-chaired due to neurologic problem. No preceding head injury or excess alcohol intake. Serology for HIV infection was negative. There was no history of yellowish discoloration of eyes, urine color change, or abdominal distension.

On physical examination, she was nourished, conscious, and oriented. Vital signs were within normal limits and stable. She had pink conjunctivae and nonicteric sclerae. Her lungs, heart, and abdomen examinations were unremarkable. On neurologic examination, she was oriented in time, place, and person. There were no cranial nerve abnormalities. No motor or sensory deficits occurred. Tandem walk and heel-to shin tests were poorly performed. She had dysarthria (slurred speech) and intention tremor of hands. Then, diagnosis of ataxic disorder due to multiple sclerosis was made at initial evaluation. Diagnosis of Wilson's disease was considered by the neurologist by pointing out the Kayser–Fleischer ring on both eyes, after the radiologist reported the possibility of Wilson's disease on MRI imaging.

**Figure 1:**
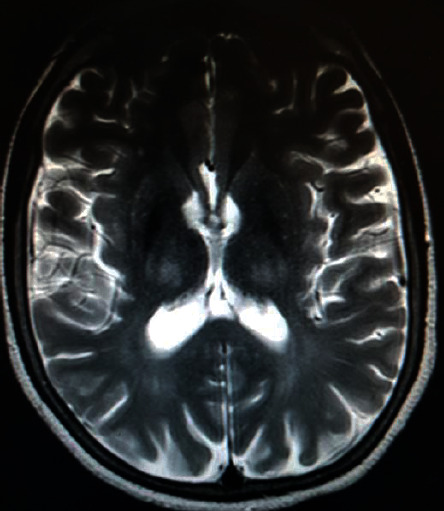
Axial T_2_-weighted MRI image showing increased signal intensity in basal ganglia and thalamus (case-1).

A laboratory examination revealed hemoglobin 15 gm/dl (normal, 12–18 gm/dl), total leukocyte count 5,200/*μ*l (normal, 4000–11,000/*μ*l; granulocyte 54%, lymphocyte 35%), platelet count 87,000/*μ*l (normal, 150,000–450,000/*μ*l), and ESR 01 mm in first hour. Serum biochemical tests were normal. Serum tests for ANA and RF were negative. The serologic test for HIV, hepatitis B, and hepatitis C infections was negative. Abdominal ultrasound examination revealed the coarse echotexture liver with surface irregularity. Axial T_2_-weighted MRI images showed bilateral hyperintensities on basal ganglia and thalamus (Figure 1). Serum ceruloplasmin level by immunoturbidimetry and 24 hr urinary copper content by inductively coupled plasma-mass spectrometry (ICP-MS) were determined in the International Diagnostic Laboratory in Addis Ababa, Ethiopia, to support the imaging diagnosis. Serum ceruloplasmin concentration was reduced (<10 mg/dl), and 24 hr urinary copper excretion was increased (142.2 *μ*g/24 hrs). In sum, the clinical signs, biochemical tests, and imaging findings met diagnostic criteria for Wilson's disease (K-F rings present, score 2; mild neurologic symptoms, score 1; serum ceruloplasmin level <10 mg/dl, score 2; urinary copper content >2 × ULN, score 2; diagnosis of Wilson's disease established if score ≥4) (Tables 1 and 2).

Global Assessment Score (GAS) for WD was determined to quantify burden of disability using domains of liver (scale 0–5), cognition and behavior (scale 0–5), motor (scale 0–5), and osseomuscular (scale 0–5) [7]. The patient scored L3C0M2O0 at time of diagnosis. Then after, she was started on D-penicillamine, 300 mg, po, daily, and dose escalated by 300 mg weekly, to reach a target dose of 900 mg, po, daily. She was as well started on zinc sulphate, 110 mg, po, twice daily, and pyridoxine 25 mg, po, daily, and advised to avoid copper-rich diet. Then, the patient was referred to the Neurology Clinic, Tikur Anbessa Hospital, Ethiopia, for close follow-up. Screening of family members for Wilson's disease was recommended.

### 2.2. Case-2

A 22-year-old, wheel-chaired, young woman presented to the University of Gondar Hospital, Northwest Ethiopia, with progressive weakness of proximal muscles (difficulty to go upstairs and stand from toilet, followed by difficulty of combing hair, and at last unable to stand) of 5-year duration and unable to keep balance of 3-year duration. She has been wheel-chaired in the last six months due to neurologic problem. She had been transiently incontinent to urine. She was treated with high-dose prednisolone (60 mg, po, daily) for six months with presumptive diagnosis of inflammatory myopathy but had no improvement. She had no memory loss or epileptic fits. Serology for HIV infection was negative. There was no history of yellowish discoloration of eyes, urine color change, or abdominal distension.

On physical examination, she was undernourished, conscious, and oriented. Vital signs were with in normal limits and stable. She had pallor of conjunctivae and nonicteric sclerae. Kayser–Fleischer rings were visible with naked eye. Her lungs, heart, and abdomen examinations were unremarkable. On neurologic examination, she was oriented in time, place, and person. No cranial nerve abnormalities occurred. She had proximal muscle weakness and atrophy on both upper and lower extremities. Deep tendon reflexes were variable. Tandem walk was difficult to perform due to weakness. She had head titubation and intention tremor of hands. Finger-to-nose was poorly performed. Diagnosis of ataxic disorder and myopathy due to Wilson's disease was considered after confirmed diagnosis of prior case. Laboratory investigations including complete blood count, biochemical tests, copper studies, and brain MRI were requested.

**Figure 2:**
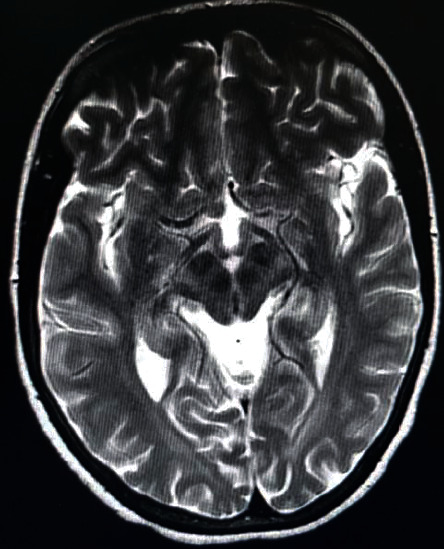
Axial T_2_-weighted MRI image showing classic “face of the giant panda” sign in midbrain hyperintensity of the tegmentum except for the red nucleus (the eyes), substantia nigra (the ears), and superior colliculus (the mouth) (case-2).

A laboratory examination revealed hemoglobin 8.6 gm/dl (normal, 12–18 gm/dl), total leukocyte count 3,700/*μ*l (normal, 4000–11,000/*μ*l; granulocyte 57%, lymphocyte 30%), and platelet count 137,000/*μ*l (normal, 150,000–450,000/*μ*l). Serum biochemical tests were normal. Serum tests for ANA and RF were negative. The serologic test for HIV, hepatitis B, and hepatitis C infections was negative. Abdominal ultrasound examination revealed the coarse echotexture liver with surface irregularity. Axial T_2_-weighted MRI images showed bilateral hyperintensities on basal ganglia, thalamus, and brain stem. “Face of the giant panda” in the midbrain was spotted on the MRI image (Figure 2). Serum ceruloplasmin level was reduced (10.5 mg/dl), and 24 hr urinary copper content was increased (110.5 *μ*g/24 hrs). In sum, the clinical signs, biochemical tests, and imaging findings met diagnostic criteria for Wilson's disease (K-F rings present, score 2; severe neurologic symptoms, score 2; serum ceruloplasmin level = 10.5 mg/dl, score 1; urinary copper content >2 × ULN, score 2; diagnosis of Wilson's disease established with score ≥4) (Tables 1 and 2). The global assessment score (GAS) was L3C0M4O4 at time of diagnosis. Then after, she was started on D-penicillamine, 300 mg, po, daily, and dose escalated by 300 mg monthly, to reach a target dose of 900 mg, po, daily. She was as well started on zinc sulphate, 110 mg, po, twice daily, and pyridoxine 25 mg, po, daily, and advised to avoid copper-rich diet. Then, the patient was referred to the Neurology Clinic, Tikur Anbessa Hospital, Ethiopia, for close follow-up.

## 3. Discussion

Wilson's disease (WD) is an inherited autosomal recessive disorder characterized by impaired hepatocellular copper metabolism. Mutation of ATP7B gene at chromosome 13 encodes defective hepatic copper transporting P-type ATPase at trans-Golgi network and cytoplasmic vesicles, which hinders ceruloplasmin synthesis and biliary excretion of copper. Hence, excess serum nonceruloplasmin bound copper results in tissue toxicity via oxidative stress and cellular apoptosis in hepatic and extrahepatic organs [2–4].

Clinical characteristics of reported Wilson's disease in Sub-Saharan Africa in the last three decades are shown in Table 3. Patients with WD initially present with a variable degree of hepatic (40–50%), neurologic (50–60%), and ophthalmic (50–90%) manifestations. Mean age of onset for hepatic disease is 10–15 years and unusual beyond 40 years. The clinical spectrum of hepatic disease may assume several forms. The asymptomatic state with persistently elevated serum transaminase activity has been witnessed. Presence of Coomb's negative hemolytic anemia, hepatic dysfunction with jaundice, ascites, coagulopathy, elevated serum transaminase activity, and hyperbilirubinemia heralds acute hepatitis. Progressive cirrhosis is a frequent mode of hepatic presentation of WD. Mean age of onset for neurologic disease is often a decade later than hepatic disease. Most patients with neurological symptoms have some degree of liver disease. The spectrum of neurological syndromes comprises Parkinsonian-type (bradykinesia, rigidity, and cognitive deficit), pseudosclerotic-type (tremor, ataxia, and dysarthria), and arrhythmic-hyperkinesia-type (chorea, athetosis, and dystonia). Tremor and dysarthria are the most common initial neurologic symptoms. Dystonia and gait disorders are often reported. A variety of psychiatric problems were documented in WD. Inappropriate behaviors include labile mood, irritability, impulsiveness, and disinhibition. Antisocial or criminal behaviors have been reported. Severe depression with suicidal attempts and frank psychosis were observed as major psychiatric disorder. Cognitive changes manifest with reduced scholastic performance, executive abilities, and visuospatial processing. Kayser–Fleischer rings and sunflower cataracts are ocular hallmarks of WD. Few of the extra hepatolenticular features consist of neuropathy, myopathy, renal tubulopathy, premature osteoporosis, and arthritis. Biochemical findings in WD consist of low serum ceruloplasmin level and increased urinary copper content. Axial T_2_-weighted and FLAIR-MRI images suggest bilateral hyperintensities on basal ganglia, brain stem, and cerebellum. “Face of the giant panda” in the midbrain is classic MRI sign [2–11]. Diagnosis of Wilson's disease is established by the scoring system proposed by “8^th^ International Meeting on Wilson Disease and Menkes Disease, Leipzig (2001),” which encompasses the combination of clinical signs, biochemical parameters, histologic findings, and mutation analysis of ATP7B gene [6].

Our patients presented with pseudosclerotic-type neurologic deficit, ocular Kayser–Fleischer rings, ultrasound-evidenced cirrhotic liver disease, axial T2-weighted MRI-hyperintensities on basal ganglia and brainstem, and abnormal copper studies (low ceruloplasmin level and increased urinary copper content), which established diagnosis of Wilson's disease. The most likely differential diagnosis for pseudosclerotic-type Wilson's disease includes Friedreich ataxia, multiple sclerosis, and manganese storage disease. Features of progressive limb and gait ataxia, dysmetria, dysarthria, and motor weakness at young age favored the autosomal recessive inherited ataxic disorder, Friedreich ataxia. Genetic testing for *frataxin*, containing expanded GAA triplet repeats, confirms the diagnosis. The other likely diagnosis was multiple sclerosis, as patients presented with gait ataxia, intention tremor, and slurred speech due to inflammatory, demyelinating cerebellar pathways. Presence of MRI-evidenced plaques in the brain and spinal cord, which disseminate in time and space, supports the diagnosis. Manganese storage disease, autosomal recessive disease, is named as “new Wilson disease” as it often presents with pseudosclerotic- or Parkinsonian-type neurological disorders and neuropsychiatric manifestations. Bilateral hyperintensities in basal ganglia is spotted in T1-weighted MRI imaging. Genetic testing for *SLC30A10* gene mutation settles the diagnosis [8].

Medical therapy such as chelators (penicillamine and trientine) and zinc salts effectively controls symptoms of WD via decoppering effect and enterocytic metalothionein-inductance, respectively. Liver transplantation is indicated in those with fulminant hepatic failure (Revised King's Score ≥11), progressive hepatic dysfunction due to advanced hepatic cirrhosis, and severe progressive neurologic deficit unresponsive to chelation therapy. Majority of patients with WD succumb from complications of progressive liver disease. Screening of family members of the index case should be routinely practiced to identify and treat early symptomatic and asymptomatic cases [2–5, 10–14].

In conclusion, Wilson's disease, declared to be an orphan disease, requires clinical acumen of physicians and expensive investigation modalities for prompt recognition and is inaccessible as required, lifelong drugs for treatment.

## Figures and Tables

**Table 1:** Laboratory results of case-1 and case-2 at time of clinical evaluation at the Neurology Clinic, University of Gondar Hospital, Northwest Ethiopia.

Variable	Case-1	Case-2	Ref. value

Complete blood count			
Hemoglobin (gm/dl)	15.2	8.6	12–18
WBC (×10^3^/*μ*l)	5.2	3.7	4–11
Platelets (×10^3^/*μ*l)	87.0	137	150–450
ESR (mm/hr)	1.0		0–20

Liver biochemical tests			
ALT (U/L)	30.0	21	5–40
AST (U/L)	41.0	40	5–40
Alkaline phosphatase (U/L)	143.0	140	50–250
Bilirubin (total) (mg/dl)	0.80	1.24	0–3–1.5
Bilirubin (direct) (mg/dl)	0.28	0.77	0–0.3
Serum total protein (gm/dl)	7.25	6.2	6–8
Serum albumin (gm/dl)	4.10	3.6	3.5–5

Coagulation profile tests			
Prothrombin time (sec)	13.5	14.6	12–14
Partial thromboplastin time (sec)	29	34	25–35
INR	1.12	1.24	0.8–1.4

Renal function tests			
BUN (mg/dl)	26.0	12	15–50
Serum creatinine (mg/dl)	1.07	0.57	0.6–1.2

Thyroid function tests			
Free T4 (ng/dl)	1.14	1.18	0.8–1.8
TSH (mIU/ml)	0.86	0.34	0.35–5.50

Copper studies			
Serum ceruloplasmin (mg/dl)	<10.0	10.5	20–60
24 hr urinary copper (*μ*g/24 hrs)	142.2	110.5	3.0–50.0

Serum electrolytes			
Potassium (meq/L)	3.9	3.6	3.5–5.0
Sodium (meq/L)	146	140	135–145
Chloride (meq/L)	112	110	102–109

Other tests			
Serum ANA	Negative	Negative	
Serum RF	Negative	Negative	
HBsAg	Negative	Negative	
Anti-HCV antibody	Negative	Negative	
Anti-HIV antibody	Negative	Negative	

**Table 2:** Diagnostic scoring system for Wilson's disease proposed by “8^th^ International Meeting on Wilson Disease and Menkes Disease, Leipzig (2001).”

Kayser–Fleischer rings	
Present	2
Absent	0

Neurologic symptoms (or typical brain MRI)	
Severe	2
Mild	1
Absent	0

Serum ceruloplasmin	
Normal (>20 mg/dl)	0
10–20 mg/dl	1
<10 mg/dl	2

Coomb's negative hemolytic anemia	
Present	1
Absent	0

Liver copper (in absence of cholestasis)	
>250 *μ*g/g	2
50–250 *μ*g/g	1
Normal (<50 *μ*g/g)	0

Urinary copper (in absence of acute hepatitis)	
Normal	0
1-2 × ULN	1
>2 × ULN	2
Normal, but >5 × ULN after penicillamine	2

Mutation analysis	
Two chromosome mutations	4
One chromosome mutation	1
No chromosomes detected	0

Total score	Evaluation
4 or more	Diagnosis established
3	Diagnosis possible, more tests needed
2 or less	Diagnosis very unlikely

Note: ULN, upper limit of normal.

**Table 3:** Summary of Wilson's disease reported in Sub-Saharan Africa in the last three decades.

Country	Age (yrs)	Sex	Diagnosis	Therapy	Publication details	Publication year

Nigeria	15	Female	Arrhythmic-hyperkynesia-type	Zinc low-copper diet	Longe et al., Arch Neurol	1982
Nigeria	8	Male	hepatic cirrhosis and Parkinsonian-type	Zinc low-copper diet	Esazobar et al., J Med Case Rep	2012
Ethiopia	15	Male	Hepatic cirrhosis	Zinc low-copper diet	Haftu et al., Case Rep Hepatol	2020

## Abbreviations

ATPase: Adenosine 5′ triphosphatase
ATP: Adenosine triphosphate
ALT: Alanine aminotransferase
AST: Aspartate aminotransferase
ANA: Antinuclear antibody
BUN: Blood urea nitrogen
ESR: Erythrocyte sedimentation rate
FLAIR: Fluid-attenuated inversion recovery
HIV: Human immunodeficiency virus
HBsAg: Hepatitis B surface antigen
MRI: Magnetic resonance imaging
RF: Rheumatoid factor
ULN: Upper limit of normal
WD: Wilson's disease.
